# Capturing the Long-Sought Dy@*C_2v_*(5)-C_80_ via Benzyl Radical Stabilization

**DOI:** 10.3390/nano12193291

**Published:** 2022-09-22

**Authors:** Xinyi Han, Jinpeng Xin, Yangrong Yao, Zhihui Liang, Yongfu Qiu, Muqing Chen, Shangfeng Yang

**Affiliations:** 1 School of Environment and Civil Engineering, Dongguan University of Technology, Dongguan 523808, China; 2 Hefei National Laboratory for Physical Sciences at Microscale, CAS Key Laboratory of Materials for Energy Conversion, Anhui Laboratory of Advanced Photon Science and Technology, Department of Materials Science and Engineering, University of Science and Technology of China, Hefei 230026, China

**Keywords:** endohedral metallofullerenes, missing fullerene, Dy@*C_2v_*(5)-C_80_, crystal structure, benzyl radical

## Abstract

Endohedral metallofullerenes (EMFs) are one type of intriguing metal/carbon hybrid molecule with the molecule configuration of sphere cavity-encapsulating metal ions/metal clusters due to their unique physicochemical properties and corresponding application in the fields of biological materials, single molecule magnet materials and energy conversion materials. Although the EMF family is growing, and versatile EMFs have been successfully synthesized and confirmed using crystal structures, some expected EMF members have not been observed using the conventional fullerene separation and purify strategy. These missing EMFs raise an interesting scientific issue as to whether this is due to the difficulty in separating them from the in situ formed carbon soot. Herein, we successfully captured a long-sought dysprosium-based EMF bearing a *C_2v_*(5)-C_80_ cage (Dy@*C_2v_*(5)-C_80_) in the form of Dy@*C_2v_*(5)-C_80_(CH_2_Ph)(Ph = −C_6_H_5_) from carbon soot containing versatile EMFs using simple benzyl radical functionalization and unambiguously confirmed the molecule structure using single crystal X-ray diffraction characterization. Meanwhile, the crystal structure of Dy@*C_2v_*(5)-C_80_(CH_2_Ph) showed that a single benzyl group was grafted onto the (5,6,6)-carbon, suggesting the open-shell electronic configuration of Dy@*C_2v_*(5)-C_80_. The theoretical calculations unveiled that the benzyl radical addition enables the modulation of the electronic configuration of Dy@*C_2v_*(5)-C_80_ and the corresponding stabilization of Dy@*C_2v_*(5)-C_80_ in conventional organic solvents. This facile stabilization strategy via benzyl radical addition exhibits the considerable capability to capture these missing EMFs, with the benefit of enriching the endohedral fullerene family.

## 1. Introduction

Endohedral metallofullerenes (EMFs) featuring versatile metallic ions/metallic clusters encapsulated in a spherical cavity have been attracting considerable interest due to the fact that they not only act as a metal/carbon hybrid molecule model to explore the structure-properties relationship but also provide a series of novel nanomaterials which can be applied in the fields of catalysts, biomaterials and energy conversion devices [1,2,3]. For instance, the non-IPR Sc_3_N@*D*_3_(6140)-C_68_ was reported to possess superior hydrogen evolution reaction (HER) performance with an onset potential of −38 mV vs. RHE due to fused pentagon rings, representing a new and promising HER catalytic motif [4]. Furthermore, the most conventional EMF, such as Sc_3_N@C_80,_ was used as ionic dopant by accepting transfer electrons from spiro-OMeTAD, leading to drastically improved conductivity and a lower Fermi level of the hole transfer layer (HTL) to minimize the Schottky barrier. As a result, the perovskite solar cells based on Sc_3_N@C_8_/spiro-OMeTAD HTL exhibit an energy conversion efficiency of 20.77% (with the champion cell exhibiting 21.09%) and improved device stability [5]. With regards to fullerene derivatives, they have been applied as electron transport layers within perovskite solar cells or organic solar cells [6,7], enabling improved device performance and suggesting the enormous prospect of EMF-derivatives in the field of energy conversion devices. Another EMF, such as Gd_3_N@C_80_ modified by oligoethylene glycol groups, exhibits enhanced magnetic resonance imaging contrast properties [2]. Since the first EMF, such as La@C_82_ reported in 1991 [8], the EMF family has been growing and was expanded to carbide clusterfullerenes [9,10], trimetallic nitrogen clusterfullerenes [11], sulfur/oxygen metallic clusterfullerenes [12,13] and so on. However, according to theoretical predications regarding possible EMFs, only a few of them are being observed or confirmed using NMR or single crystal characterization. For example, EMFs with a C_80_ cage obeying the isolated pentagon rule (IPR) were proposed to have seven types of isomeric structure including *D_5d_*, *D_2_*, *C_2v_*(3), *D_3_*, C*_2v_*(5), *D_5h_* and *I_h_* based on theoretical predictions, while the observed or confirmed species were only *C_2v_*(3), *C_2v_*(5), *D_5h_* and *I_h_* using experimental approaches. The stable formation of EMFs was attributed to the synergistic influence of the transferred charge number from the endohedral metallic ions/clusters to the outer fullerene cages as well as the matched energy levels between the inner metals/clusters and outer fullerene cages [14]. For example, *I_h_*-C_80_ can be stabilized by accepting six electrons from the encapsulating metal ions/metallic clusters including M_3_N clusters (M = Sc, Y, La, Ce, Pr, Nd, Gd, Tb, Dy, Ho, Er, Tm, Lu) and M_2_TiC clusters (M = Sc, Y, Tb, Dy, Lu) [15]. When anomalous five-electron transfer occurs on M_2_@*I_h_*-C_80_ (M = Y_2_, Gd_2_, Tb_2_, Dy_2_, Ho_2_, Er_2_, Tb_2_ and TbGd) [16,17], a benzyl radical was verified to be the efficient approach to stabilize the missing M_2_@*I_h_*-C_80_ using bromide benzyl in mild experimental conditions [18]. 

With regards to EMFs with a *C_2v_*(3)-C_80_ cage, the two-electron transfer from the endohedral divalent metal ions to the *C_2v_*(3)-C_80_ cage enable the stabilization of M^2+^@*C_2v_*(3)-C_80_^2−^ (M = Sm, Yb) [19,20]. However, with the trivalent rare earth metal ions encapsulated within the *C_2v_*(3)-C_80_ cage along with the three-electron transfer, the corresponding M^3+^@*C_2v_*(3)-C_80_^3−^ is difficult to stabilize and obtain only if chemical modification is adopted to stabilize them [21,22]. The first and only case of missing M^3+^@*C_2v_*(3)-C_80_^3−^ is La^3+^@*C_2v_*(3)-C_80_^3−^ reported by Nagase et al., showing that the dichlorobenzene radical formed in situ from the 1, 2, 4-trichlorobenzene solution under thermal conditions enables the stabilization of La^3+^@*C_2v_*(3)-C_80_^3−^ in the form of La@C_80_(C_6_H_3_Cl_2_). The theoretical calculations revealed that La@*C_2v_*(3)-C_80_ is an open-shell electron configuration and has high reactivity resulting from its small ionization potential (Ip) and electron affinity (E_a_), which is efficiently stabilized by the addition of the dichlorobenzene radical [23]. In addition, EMFs with a *C_2v_*(5)-C_80_ carbon cage can be stabilized by accepting four electrons from the endohedral carbide clusters (Sc_2_C_2_, Er_2_C_2_) or oxygen clusters (Sc_2_O, Lu_2_O) [24,25,26,27]. Considering the missing La^3+^@ *C_2v_*(3)-C_80_^3−^, it was stabilized with three-electron transfer and entrapped in the form of La@C*_2v_*(3)-C_80_(C_6_H_3_Cl_2_) using facile radical addition. Therefore, whether the long-sought monometallic M^3+^@*C_2v_*(5)-C_80_^3−^ (M = Sc, Y, Ce, Pr, Nd, Gd, Tb, Dy, Ho, Er, Tm, Lu) can be stabilized and entrapped using facial chemical functionalization is an interesting issue. To address the issue mentioned above, we performed a study searching for the missing M^3+^@*C_2v_*(5)-C_80_^3−^ (M = rare earth metals) EMFs using the facile benzyl radical stabilization strategy. 

Herein, we adopted the freshly prepared dysprosium (Dy)-based raw soot as the research target and successfully captured the missing Dy@C_80_ in the existing form of Dy@C_80_(C_7_H_7_). More importantly, the molecular structure of Dy@C_80_ was unambiguously confirmed using single crystal X-ray diffraction characterization, showing that the C_80_ cage is a *C_2v_*(5) symmetry and the addition site is on a pentagon–hexagon–hexagon junction ([5,6,6]-junction) carbon atom. Dy@*C_2v_*(5)-C_80_ is the first reported case of a monometallic EMF isomer composed of trivalent M^3+^ (M = rare earth metals) and a *C_2v_*(5)-C_80_ carbon cage. The theoretical calculations indicated that the benzyl radical addition clearly alters the energy level and enlarges the bandgap, leading to the stabilization of Dy@*C_2v_*(5)-C_80_. 

## 2. Experiment

### 2.1. Characterization Techniques

Soot containing Dy-EMFs was produced using a modified Krätschmer–Huffman DC-arc discharge method, and after the radical addition, the extracted EMF derivatives were isolated and purified using high performance liquid chromatography (HPLC, LC-9104, Japan Analytical Industry, Akishima, Japan)). Regarding the Dy@C_80_(CH_2_Ph), the corresponding purification was performed using a multistep HPLC separation procedure with toluene as the eluent. The pure Dy@C_80_(CH_2_Ph) was further verified using analytical HPLC and matrix-assisted laser desorption ionization time-of-flight mass spectrum (MALDI-TOF MS) with 1,1,4,4-tetraphenyl-1,3-butadiene as a matrix (Biflex III, Bruker Daltonics Inc., Germany). Absorption spectrum of Dy@C_80_(CH_2_Ph) in toluene was recorded on a UV−vis−NIR 3600 spectrometer (Shimadzu, Kyoto, Japan) using a quartz cell of 1 mm thickness. Electrochemical studies of Dy@C_80_(CH_2_Ph) were performed in o-DCB (anhydrous, 99%, Aldrich, St. Louis, MI, USA). The supporting electrolyte was TBAPF_6_ (electrochemical grade, Fluka) which was dried under reduced pressure at 340 K for 24 h and stored in glovebox. Cyclic voltammogram experiments were performed using a CHI660D electrochemical workstation (CHI Instrument, Austin, TX, USA) at room temperature. A standard three-electrode arrangement including a platinum disc as working electrode, a platinum wire as counter electrode and a silver wire as a reference electrode was used. In a control experiment, ferrocene (Fc) was added as the external standard, and all potentials were referred to as Fc/Fc^+^ couple.

### 2.2. The Capturing and Separation of Dy@C_80_(CH_2_Ph)

The composite rods of graphite and Dy_2_O_3_ (molar ratio of Dy:C = 1:15) were evaporated using DC-arc discharge method under a 180 mbar He atmosphere and 110A DC, delivering the raw soot containing a series of Dy-EMFs. Before entrapping the missing Dy@C_80_ derivative, the collected freshly prepared carbon soot was treated with N,N-Dimethylformamide (DMF) due to its reduction properties, enabling the missing EMFs to dissolve in solution through transferring electrons as per the same strategy applied in M_2_@*I_h_*-C_80_ (M = Y, Gd, Tb, Dy, Ho, Er, Dy, Tb, Gd), as previously reported [16,17]. In detail, the raw soot obtained from arc discharge procedure was dispersed in DMF and purged using nitrogen for 20 min, and then the mixture solution was heated to 150 °C for 20 h under nitrogen atmosphere. Then, the solution temperature was decreased to 110 °C, and benzyl bromide was injected into the mixture solution via syringe to avoid the entrance of air. The radical addition reaction occurred, and the corresponding reaction process lasted 20 h under 110 °C. When the reaction finished, the reaction solution was cooled to room temperature, and the insoluble solid mixture was filtrated using reduced pressure. The obtained DMF solution containing pristine and functionalized EMFs was dried to remove DMF solvent using a rotary evaporator, and the residual black solid was washed with methanol. Finally, the solid was dissolved in toluene for further isolation using multistep HPLC procedure.

### 2.3. Crystal Growth and Measurements

The black crystals of Dy@C_80_(CH_2_Ph) suitable for single crystal X-ray diffraction measurements were obtained using the slow volatilization of toluene/CS_2_ solution containing Dy@C_80_(CH_2_Ph) and decapyrrylcorannulene (DPC) as host for about two weeks [28]. The black crystals were subjected to the Shanghai Synchrotron Radiation Facility (beamline station BL17B at 100 K) for crystal data collection. The crystal structure was refined using all data (based on F^2^) utilizing SHELXL 2015 within OLEX2. The corresponding crystal data can be obtained free of charge from The Cambridge Crystallographic Data Centre (CCDC numbers: 2195196).

## 3. Results and Discussion

### 3.1. Stabilization, Separation and Purification of Dy@C_80_ Derivative

**Scheme 1:**
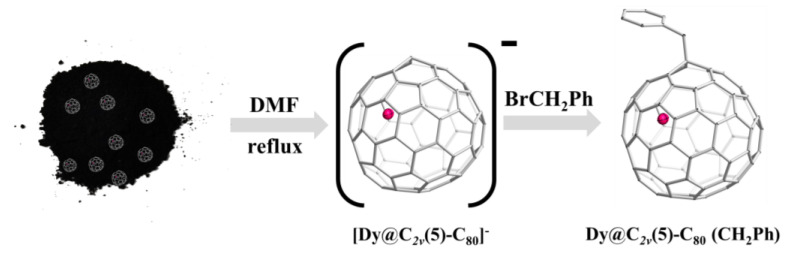
The activation and stabilization of missing Dy@*C_2v_*(5)-C_80._

**Figure 1:**
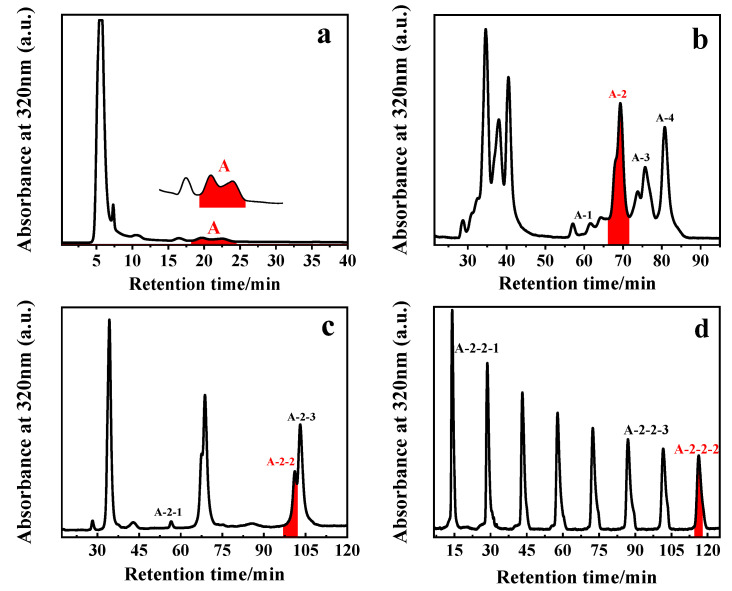
Isolation and purification of Dy@C_80_(CH_2_Ph) via four-step HPLC process. (**a**) Collected fraction A contained Dy@C_80_(CH_2_Ph) and labelled as the red region. Conditions: Buckyprep column (ø 10 × 250 mm), a flow rate of 4 mL/min, injection volume of 4 mL, toluene as eluent at 40 °C. (**b**) Recycling HPLC profile of fraction A for fraction A-2. Conditions: Buckyprep column (ø 20 × 250 mm), a flow rate of 8 mL/min, injection volume of 8 mL, toluene as eluent at 40 °C. (**c**) Recycling HPLC profile of fraction A-2 for A-2-2. Conditions: Buckyprep column (10 × 250 mm), a flow rate of 4 mL/min, injection volume of 4 mL, toluene as eluent at 40 °C. (**d**) HPLC profile of fraction A-2-2 for consequence A-2-2-2. Conditions: Buckyprep-M column (10 × 250 mm), a flow rate of 4 mL/min, injection volume of 4 mL, toluene as eluent at 40 °C.

**Figure 2:**
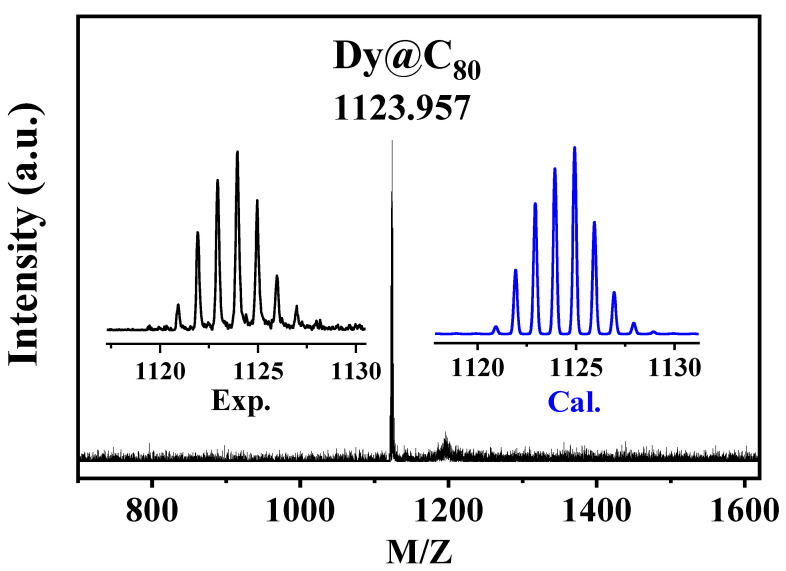
MALDI-TOF MS of the fraction A-2-2-2 with 1,1,4,4-tetraphenyl-1,3-butadiene as matrix.

Soot containing Dy-EMFs was produced using the DC-arc discharge method under an He atmosphere, which thereafter was dispersed in acetone and subjected to MALDI-TOF mass spectrometry for the initial confirmation of empty fullerene and EMFs. Surprisingly, a mass spectrum peak at *m/z* = 1124.28 was observed and was assigned as Dy@C_80_, indicating that the long-sought Dy@C_80_ had been hidden in the carbon soot (Figure S1). However, the pure Dy@C_80_ and corresponding structure confirmation have never been reported. The missing Dy@C_80_ is similar to M_2_@*I_h_*-C_80_ (M = Y, Gd, Tb, Dy, Ho, Er, Dy, Tb, Gd) as it is difficult to stabilize in conventional organic solvents [16,17]; therefore, whether the benzyl radical addition to Dy@C_80_ enables the efficient trapping and stabilization of Dy@C_80_ in the form of Dy@C_80_(CH_2_Ph) is in question. It is noteworthy that the critical step to entrap the missing Dy@C_80_ is the ionization of Dy@C_80_ using DMF reduction as showed in Scheme 1 similar to M_2_@*I_h_*-C_80,_ as discussed above [16,17]. More importantly, in contrast to the pristine EMFs with regular retention time in HPLC profiles, the benzyl radical EMFs greatly shorten their retention time in HPLC profiles. Therefore, the first important separation step to Dy@C_80_(CH_2_Ph) was to determine its retention time location in an HPLC profile for fraction collection and further purification, which was verified using MALDI-TOF MS. The collected fraction A, through the HPLC isolation process in Figure 1a, was characterized using MALDI-TOF MS. This showed that the typical molecular ion peak of Dy@C_80_(CH_2_Ph) at *m/z* = 1124.28 was clearly observed the same as the pristine Dy@C_80,_ which was attributed to the dissociated benzyl group under laser irradiation (Figure S2). Apart from the Dy@C_80_ derivative, two other intensive molecular ion peaks at 1148.38 and 1286.30 assigned to Dy@C_82_ and Dy_2_@C_80_ derivatives, respectively, indicate the requirement of further HPLC isolation to exclude these two EMF derivatives. After a four-step HPLC separation procedure, we successfully obtained the pure Dy@C_80_ derivative which was further verified using analytical HPLC (Figure S3) and MALDI-TOF mass spectroscopy (Figure 2). 

### 3.2. Molecular Structure Confirmation of Dy@C_80_(CH_2_Ph)

HPLC analysis and mass spectroscopy characterization only provide an ambiguous structure in which the carbon cage symmetry, metallic ion location and addition site regioselectivity are not yet clear. Therefore, the single crystal structure of Dy@C_80_(CH_2_Ph) is critical for unveiling its precise molecule structure and corresponding physiochemical properties. Fortunately, black crystals suitable for the single crystal measurements were obtained using the slow volatilization of toluene/CS_2_ solution containing Dy@C_80_(CH_2_Ph) and DPC as hosts. The crystal structure of Dy@C_80_(CH_2_Ph) was unambiguously confirmed using single crystal X-ray diffraction measurements, showing that this crystal is composed of two DPC molecules and one Dy@C_80_(CH_2_Ph), and the detailed crystal data are summarized in Table S1. 

**Figure 3:**
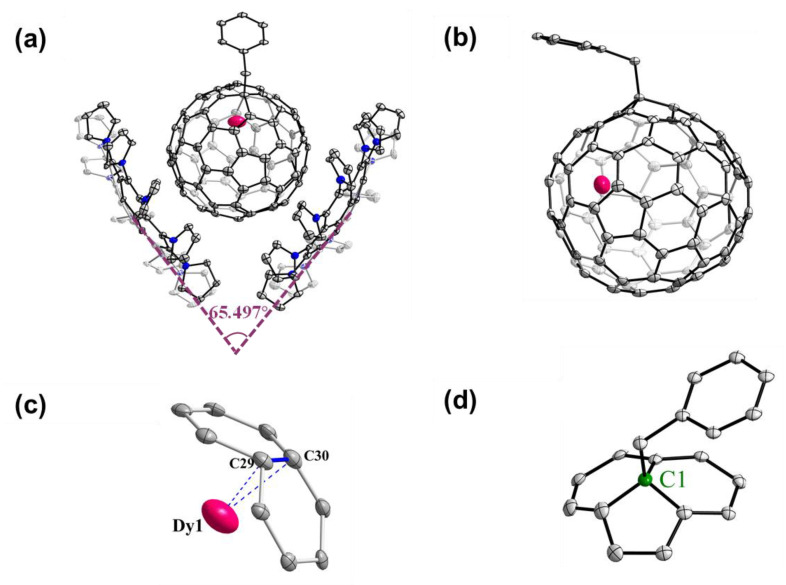
Drawing of (**a**) Dy@*C_2v_*(5)-C_80_(CH_2_Ph)·2DPC and (**b**) Dy@*C_2v_*(5)-C_80_(CH_2_Ph). (**c**) Fragment view showing the interaction of the major Dy1 with the closest carbon atoms of the cage and (**d**) the addition site of benzyl. Only major Dy site is shown. Solvent molecules and H atoms are omitted for clarity. Gray: C; blue: N; fuchsia: Dy.

The relative orientation of the Dy@C_80_(CH_2_Ph) and two DPC molecules is shown in Figure 3a, in which the two DPC molecules present a V-shape geometry with the dihedral angle of 65.5°. In contrast to the pristine Sc_3_N@*I_h_*-C_80_·2DPC complex with a dihedral angle of 1.49°, the enlarged dihedral angle of Dy@C_80_(CH_2_Ph) was perhaps attributable to the steric hindrance of the grafted benzyl group. Furthermore, the crystal structure of Dy@C_80_(CH_2_Ph) shows that one benzyl group was grafted onto the fullerene cage with the addition site of the [5,6,6]-carbon (Figure 3b,d). The detailed analysis of the carbon cage indicates that the symmetry of C_80_ is *C_2v_*(5), which is the first case of an EMF composed of a trivalent rare earth metal ion and *C_2v_*(5)-C_80_ carbon cage. 

Regarding the endohedral Dy^3+^ ion, there are four position disorders in the *C_2v_*(5)-C_80_ cage with the occupancy ratio of Dy1 0.79, Dy2 0.10, Dy3 0.06 and Dy4 0.05, respectively (as seen in Figure S4 and Table S1). The major occupancy Dy1 is located underneath the [6,6]-bond (the bond shared by two hexagonal rings) with the shortest distances of 2.250(13) Å (Dy1-C30) and 2.236(13) Å (Dy1-C29) between Dy1 and the adjacent carbon atoms (Figure 3c), respectively, and indicates the strong interaction between the endohedral metal ion and the outer *C_2v_*(5)-C_80_. Conventionally, the encapsulated metal ion is located at the symmetric plane or the symmetric center of the EMF cages [29], and the main occupancy of endohedral Dy1 clearly deviated from the normal position of pristine Dy@*C_2v_*(5)-C_80_ after the benzyl functionalization, indicating the remarkable electron configuration change in Dy@*C_2v_*(5)-C_80_(CH_2_Ph) (Figure S5). 

**Figure 4:**
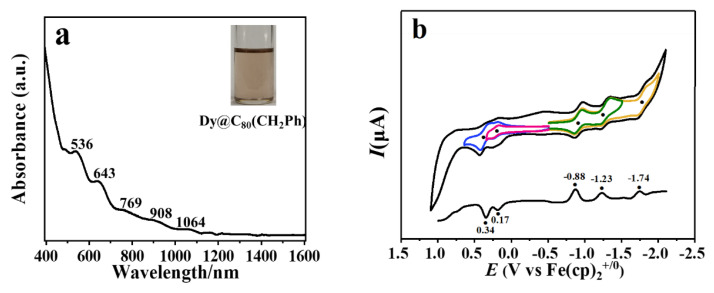
(**a**) UV-Vis-NIR absorption spectrum of Dy@*C_2v_*(5)-C_80_(CH_2_Ph). (**b**) Cyclic voltammograms of Dy@*C_2v_*(5)-C_80_(CH_2_Ph) in o-DCB solution with ferrocene (Fc) as the standard under different scan regions. Scan rate: 100 mV/s, TBAPF_6_ as supporting electrolyte. The half-wave potentials (E_1/2_) of each redox step are marked with a solid dot to aid comparison.

To reveal the absorption and electrochemical properties of Dy@*C_2v_*(5)-C_80_(CH_2_Ph), the corresponding UV-vis-NIR absorption and cyclic voltammetry measurements were performed, as shown in Figure 4a. The absorption spectrum of Dy@*C_2v_*(5)-C_80_(CH_2_Ph) in toluene exhibits six obvious absorption peaks at 489, 536, 643, 769, 908 and 1064 nm. The absorption onset at 1123 nm suggests that the calculated optical bandgap (ΔE_gap, optical_) of Dy@*C_2v_*(5)-C_80_(CH_2_Ph) is 1.10 eV. Compared with the absorption profile of *C_2v_*(5)-C_80_ embedding Sc_2_C_2_, Sc_2_O, Er_2_C_2_ and Lu_2_O clusters with four-electron transfer to the outer carbon cage, Dy@*C_2v_*(5)-C_80_(CH_2_Ph) exhibits a remarkable absorption difference which is attributed to the three-electron transfer and benzyl radical addition. The electrochemical characterization of Dy@*C_2v_*(5)-C_80_(CH_2_Ph) was obtained using cyclic voltammograms with tetrabutylammonium hexafluorophosphate (TBAPF_6_) as the electrolyte in o-dichlorobenzene (o-DCB). The redox potentials of Dy@*C_2v_*(5)-C_80_(CH_2_Ph) show three reversible reduction peaks and two irreversible oxide peaks. The corresponding differential pulse voltammetry (DPV) in Figure 4b shows that the first reduction potential and first oxide potential are −0.88 V and 0.17 V, respectively, delivering a narrow bandgap of 1.05 eV. To understand the redox change before and after the benzyl radical addition of Dy@*C_2v_*(5)-C_80_, the analogous monometallic EMFs, such as La@*C_2v_*-C_82_ and La@*C_2v_*-C_82_-benzyl adduct, were applied to analogize the variation tendency of electrochemical properties. After the benzyl radical modification, the first oxide and reduction potentials of La@*C_2v_*-C_82_ at 0.07 V and -0.42 V positively shift about 0.10~0.63 V to 0.15~0.25 V for the first oxide potential and -1.05~-0.68 V for the first reduction potential. According to this change tendency, the first oxide potential of Dy@*C_2v_*(5)-C_80_ is perhaps close to zero potential, even approaching the negative value, which is possibly responsible for the instability of the Dy@*C_2v_*(5)-C_80_. 

**Figure 5:**
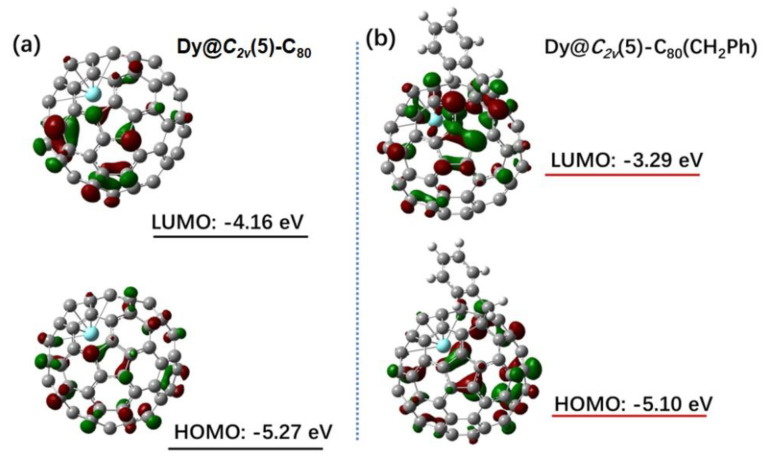
The electronic configuration (HOMO and LUMO energy levels) of (**a**) Dy@C_2v_(5)-C_80_ and (**b**) Dy@C_2v_(5)-C_80_(CH_2_Ph) using density functional theory.

The theoretical calculations of Dy@*C_2v_*(5)-C_80_ and Dy@*C_2v_*(5)-C_80_(CH_2_Ph) were performed, as shown in Figure 5. The highest occupied molecular orbital (HOMO) energy level and lowest unoccupied molecular orbital (LUMO) energy level calculated are: −5.27 eV and -4.16 eV, respectively, for Dy@*C_2v_*(5)-C_80_; and −5.10 eV and −3.29 eV, respectively, for Dy@*C_2v_*(5)-C_80_(CH_2_Ph). The corresponding bandgap for Dy@*C_2v_*(5)-C_80_ and Dy@*C_2v_*(5)-C_80_(CH_2_Ph) are 1.11 eV and 1.86 eV, respectively. The change tendency of LUMO, HOMO energy levels and bandgap for Dy@*C_2v_*(5)-C_80_ and Dy@*C_2v_*(5)-C_80_(CH_2_Ph) indicate that the benzyl radical addition enlarges the bandgap from 1.11 eV to 1.86 eV and elevates the LUMO energy level to about 0.87 eV which is beneficial for the stabilization of the missing Dy@*C_2v_*-C_80_.

## 4. Conclusions

The long-sought Dy@*C_2v_*-C_80_ was successfully trapped in the form of Dy@*C_2v_*(5)-C_80_(CH_2_Ph) using a facile reduction and radical addition strategy. After a four-step HPLC isolation procedure, the pure Dy@*C_2v_*(5)-C_80_(CH_2_Ph) was obtained and unambiguously confirmed using single crystal X-ray diffraction measurements. The crystal structure of Dy@*C_2v_*(5)-C_80_(CH_2_Ph) shows that one benzyl group was grafted onto the [5,6,6]-carbon atom, suggesting that the Dy@*C_2v_*(5)-C_80_ is an open-shell electron configuration and a three-electron transfer configuration of Dy^3+^@*C_2v_*(5)-C_80_^3−^. More importantly, the carbon cage symmetry was unveiled to be a rare *C_2v_*(5)-C_80_ which is the first case of an EMF composed of a monometal rare earth ion encapsulated within a C_80_ cage. Meanwhile, the encapsulated Dy^3+^ ion of Dy@*C_2v_*(5)-C_80_(CH_2_Ph) is located underneath the [6,6]-bond and is deviated from the symmetry plan of *C_2v_*(5)-C_80_, indicating the remarkable electron configuration change after the benzyl radical addition. A cyclic voltammogram of Dy@*C_2v_*(5)-C_80_(CH_2_Ph) shows that the narrow bandgap of 1.10 eV is responsible for the instability of Dy@*C_2v_*(5)-C_80_. The theoretical calculations showed that benzyl radical addition to Dy@*C_2v_*(5)-C_80_ enlarges the bandgap from 1.11 eV to 1.86 eV and the clearly elevated LUMO energy level to about 0.87 eV, which synergistically stabilizes the missing Dy@*C_2v_*(5)-C_80_. Therefore, the benzyl radical strategy was verified as an efficient chemical method to trap some long-sought EMFs proposed by theoretical predications, and there may be benefits to exploring the novel EMFs hidden within raw soot.

## Data Availability

The data presented in this study are available on request from the corresponding author. Meanwhile, the detailed crystal data can be obtained free of charge from The Cambridge Crystallographic Data Centre via www.ccdc.cam.ac.uk/data_request/cif (accessed on 5 August 2022).

## Supplementary Materials

The following supporting information can be downloaded at: https://www.mdpi.com/article/10.3390/nano12193291/s1, Figure S1. MALDI-TOF mass spectrum of raw soot containing Dy@C_80_; Figure S2. MALDI-TOF mass spectrum of fraction A with the matrix of 1,1,4,4-tetraphenyl-1,3-butadiene; Figure S3. HPLC chromatograms of purified Dy@C_80_(CH_2_Ph). Condition: Buckyprep column (ø 4.6 mm × 250 mm), UV-detector (320 nm), toluene as eluent with the flow rate of 1.0 mL/min; Figure S4. The disordered positions of dysprosium sites in Dy@*C_2v_*(5)-C_80_(CH_2_Ph). Gray: C; Fuchsia: Dy; Figure S5. Relative orientation between endohedral Dy ion and *C_2v_*(5)-C_80_ carbon cage of Dy@*C_2v_*(5)-C_80_(CH_2_Ph). Gray: C; Fuchsia: Dy; Table S1. Crystal data of Dy@*C_2v_*(5)-C_80_(CH_2_Ph); Table S2. The occupancy of disordered metal ions encapsulated within Dy@*C_2v_*(5)-C_80_(CH_2_Ph); Table S3. Redox Potentials (V vs. Fc^+^/Fc) and Electrochemical Gaps (ΔE_gap_, EC) of Dy@*C_2v_*(5)-C_80_(CH_2_Ph).
