# Supplementary material for: Capturing the Long-Sought Dy@C2v(5)-C80 via Benzyl Radical Stabilization
Source: Nanomaterials (Basel). 2022 Sep 22;12(19):3291. doi: 10.3390/nano12193291 (PMC9565622; doi:10.3390/nano12193291)
Supplement: Supplementary file 1 [file nanomaterials-12-03291-s001.zip › nanomaterials-1899985-supplementary.pdf]

nanomaterials

Supplementary Materials

Capturing the Long-sought Dy@C_{2v}(5)-C_{80} via Benzyl Radical Stabilization

Xinyi Han[1,2], Jinpeng Xin[2], Yangrong Yao[2], Zhihui Liang[1], Yongfu Qiu[1], Muqing Chen[*1,2], Shangfeng Yang[*2]

1 School of Environment and Civil Engineering, Dongguan University of Technology, Dongguan, Guangdong, 523808, China; mqchen@ustc.edu.cn (M. C.); liangzh@dgut.edu.cn (Z. L.); qiuyf1979@foxmail.com (Y. Q.)

2. Hefei National Laboratory for Physical Sciences at Microscale; CAS Key Laboratory of Materials for Energy Conversion; Anhui Laboratory of Advanced Photon Science and Technology, Department of Materials Science and Engineering, University of Science and Technology of China, Hefei 230026, Anhui, China; han1998@mail.ustc.edu.cn (X. H.); xinjinpeng@mail.ustc.edu.cn (J. X.); yryao@ustc.edu.cn(Y. Y.); mqchen@ustc.edu.cn (M. C.); sfyang@ustc.edu.cn (S. Y.)

Table of Contents

1. Experimental details………………………………………………… S1

2. Synthesis, separation and structure characterization……………….S1

3. Electrochemical measurements……….……….……..…..………..….S3

1. Experimental Details

1.1 Materials

Dy_2O_3 was purchased from Lanthanide New Materials Co., Ltd (Suzhou, China). Graphite rod were purchased from Sinosteel New Graphite Materials Co., Ltd (Shanghai, China). Dimethylformamide (DMF), Toluene and methanol were purchased from Sinopharm Chemical Reagent Co., Ltd (Shanghai, China). Benzyl bromide were purchased form Aladdin (Shanghai, China).

2. Synthesis, Separation and Structure Characterization

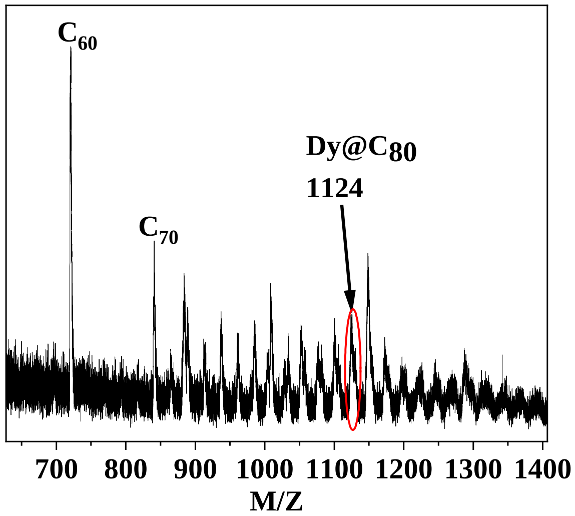

Figure S1. MALDI-TOF mass spectrum of raw soot containing Dy@C_{80}.

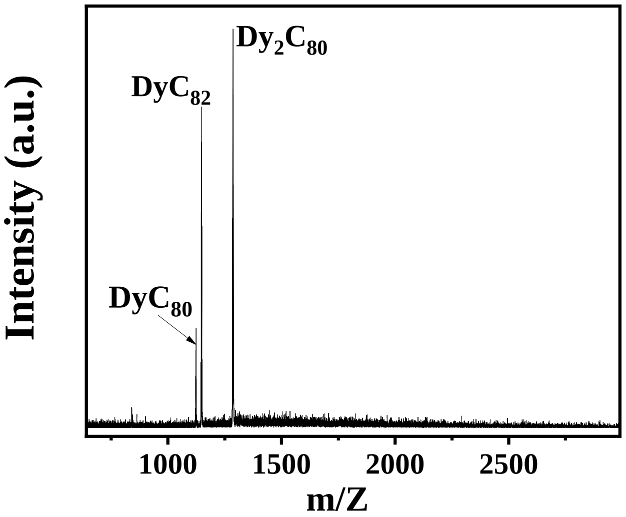

Figure S2. MALDI-TOF mass spectrum of fraction A with the matrix of 1,1,4,4-tetraphenyl-1,3-butadiene.

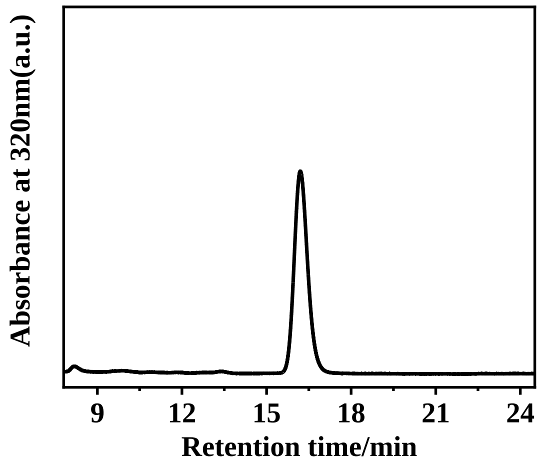

Figure S3. HPLC chromatograms of purified Dy@C_{80}(CH_2Ph). Condition: Buckyprep column (ø 4.6 mm × 250mm), UV-detector (320 nm), toluene as eluent with the flow rate of 1.0 mL/min.

Table S1. Crystal data of Dy@C_{2v}(5)-C_{80}(CH_2Ph).

Compound	2DPC{Dy@C_{2v}(5) – C_{80}(CH_2Ph)}
T, K	100(2)
Formula weight	3108.59
λ, Å	0.71073
color/habit	black/block
Empirical formula	$C_{214}H_{95}N_{20}Dy$
crystal system	monoclinic
space group	$P2_1/c$
a, Å	14.701
b, Å	32.466
c, Å	32.045
α, deg	90
β, deg	101.81
γ, deg	90
V, $Å^3$	14970.7
Z	4
ρ, g/cm^3	1.379
μ, mm^{-1}	0.569
R_1 [reflections with I>2σ(I)]	0.0913 (8046)
wR_2 (all data)	0.2585 (14703)

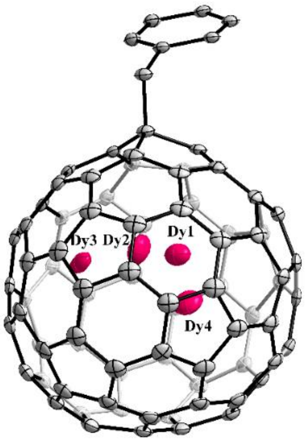

Figure S4. The disordered positions of dysprosium sites in Dy@C_{2v}(5)-C_{80}(CH_2Ph). Gray: C; Fuchsia: Dy.

Table S2. The occupancy of disordered metal ions encapsulated within Dy@C_{2v}(5)-C_{80}(CH_2Ph).

**Compounds**	**Occupancy of metal ion**			
Dy@C_{2v}(5)-C_{80}(CH_2Ph)	Dy1	Dy2	Dy3	Dy4
	0.79	0.10	0.06	0.05

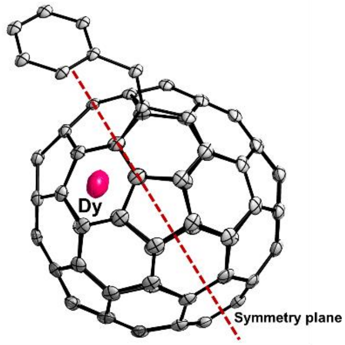

Figure S5. Relative orientation between endohedral Dy ion and C_{2v}(5)-C_{80} carbon cage of Dy@C_{2v}(5)-C_{80}(CH_2Ph). Gray: C; Fuchsia: Dy.

3. Electrochemical Measurements and Theoretical Calculation

Table S3. Redox Potentials (V vs Fc^+/Fc) and Electrochemical Gaps ($\Delta E_{gap,\ EC}$) of Dy@C_{2v}(5)-C_{80}(CH_2Ph)

**EMFs**	$^{red}E_1$	$^{red}E_2$	$^{red}E_3$	$^{ox}E_1$	$^{ox}E_2$	$\Delta E_{gap,EC}$**(V)**
Dy@C_{2v}(5)-C_{80} (CH_2Ph)	-0.88	-1.23	-1.74	0.17	0.34	1.05
